# Effects of cupping therapy for obesity: A protocol for systematic review and meta-analysis

**DOI:** 10.1097/MD.0000000000027701

**Published:** 2021-11-05

**Authors:** Hyungsuk Kim, Koh-Woon Kim, Won-Seok Chung

**Affiliations:** a Department of Korean Medicine Rehabilitation, Kyung Hee University Medical Center, 23 Kyungheedae-ro, Dongdaemun-gu, Seoul, Korea; b Department of Korean Rehabilitation Medicine, College of Korean Medicine, Kyung Hee University, Seoul, Korea.

**Keywords:** cupping therapy, meta-analysis, obesity, protocol, systematic review

## Abstract

**Background:**

Obesity is a prevalent disease in modern society. Despite the various interventions available in conventional medicine, their success rates are not satisfactory because of the complex mechanisms involved in obesity. Cupping therapy is a traditional Chinese medicinal intervention, and it has become widely used in various clinical settings for the treatment of obesity. This systematic review and meta-analysis will investigate the effects of cupping on obesity.

**Methods:**

Three Korean databases (KoreaMed, Oriental Medicine Advanced Searching Integrated System, and ScienceON), 1 Japanese database (Citation Information by the National Institute of Informatics), 1 Chinese database (Chinese National Knowledge Infrastructure), MEDLINE/PubMed, EMBASE, and The Cochrane Central Register of Controlled Trials will be searched for studies published until March 2021. The primary outcome is body weight. The secondary outcomes will be body mass index, waist-hip ratio, waist circumference, hip circumference, body fat mass, body fat percentage, and adverse events.

**Results:**

This systematic review and meta-analysis will evaluate the effects of cupping therapy for obesity.

**Conclusion:**

The results of this systematic review and meta-analysis will then be discussed in a related journal for clinicians working with obese patients to apply the interventions in this article.

**Trial registration number:**

DOI 10.17605/OSF.IO/P8JVM (https://osf.io/p8jvm).

## 1 Introduction

Obesity is a prevalent health condition in modern society. It is related to a lack of physical activities due to the development of technology and the overconsumption of food.^[1]^ Obesity is more than just the state of being overweight because it can lead to many other lethal diseases, such as cardiovascular disease^[2]^ and cancer.^[3]^

Many interventions have been applied to treat obesity. The first-line therapy is diet control and exercise. Many types of diets for reducing weight have also been suggested by healthcare researchers, such as a low-calorie diet^[4]^ or intermittent fasting.^[5]^ For exercise, both aerobic and strength exercises are recommended for better results.^[6]^ If these are not effective, medications are described by doctors. However, these dietary and locomotive interventions should be simultaneously conducted when applying drug therapy. With these therapies, the success rate is still low because the overweight condition is caused by various factors.^[7]^

Cupping therapy, a traditional Chinese medicinal intervention, is a safe physical stimulus that has been proven to be effective in musculoskeletal, dermatological, and pulmonary diseases.^[8]^ It is used widely in clinical situations for overweight in East Asian countries, and randomized controlled trials on cupping therapy for obesity and overweight have been conducted. Despite the developments in these clinical and research areas, only 1 systematic review and meta-analysis, published in a Korean journal, is available.^[9]^ The article only targeted a Chinese database, and the search period was only until 2015. As such, it does not reflect recent discoveries.

In this protocol, we present a protocol for a systematic review and meta-analysis of cupping therapy for obesity to verify its effect.

## 2 Methods

### 2.1 Study registration

This protocol is based on the preferred reporting items for systematic reviews and meta-analysis protocols.^[10]^ This protocol has been registered in the Open Science Framework (osf.io/p8jvm).

### 2.2 Eligibility criteria for study selection

#### 2.2.1 Types of studies

Only randomized controlled trials will be analyzed in this review to ensure a higher quality of evidence. Quasi- or crossover designs will not be considered. There will be no language limitations in the selection process.

#### 2.2.2 Participants

We will focus on patients diagnosed with obesity, with no restrictions on the age and sex.

#### 2.2.3 Types of interventions

The experimental group treated with cupping therapy will be included in this study. Blood-letting cupping will also be considered for analysis in this review because it also has the constituents of cupping. Control groups can be sham, no treatment, or standard conventional treatment. Cupping therapy that is accompanied by other therapies will also be included if the same kind of treatment is applied to both the experimental and control groups. Accompanied therapy can be drugs, lifestyle interventions, and other types of interventions from traditional Chinese medicine.

#### 2.2.4 Types of outcome measures

The body weight will be the primary outcome because it is the most essential outcome of obesity. Other measurements such as body mass index, waist-hip ratio, waist circumference, hip circumference, body fat mass, body fat percentage, and adverse events will be the secondary outcomes.

### 2.3 Search strategy

#### 2.3.1 Electronic data

Three Korean databases (KoreaMed, Oriental Medicine Advanced Searching Integrated System, and ScienceON), 1 Japanese database (Citation Information by the National Institute of Informatics), 1 Chinese database (Chinese National Knowledge Infrastructure), MEDLINE/PubMed, EMBASE, and The Cochrane Central Register of Controlled Trials will be searched from their inception to March 2021. The search strategy for PubMed is presented in Table 1.

**Table 1:** Search strategy for PubMed.

“obes∗”[All Fields] OR “weight gain∗”[All Fields] OR “weight loss”[All Fields] OR “body mass ind∗”[All Fields] OR “adipos∗”[All Fields] OR “overweight”[MeSH Terms] OR “overweight”[All Fields] OR “overweighted”[All Fields] OR “overweightness”[All Fields] OR “overweights”[All Fields]) OR “over weight”[All Fields] OR “overload syndrome∗”[All Fields] OR “overeat∗”[All Fields] OR “over eat∗”[All Fields] OR “overfeed∗”[All Fields] OR “over feed∗”[All Fields] OR “weight cycling”[All Fields] OR “weight reduc∗”[All Fields] OR “weight los∗”[All Fields] OR “weight maint∗”[All Fields] OR “weight decreas∗”[All Fields] OR “weight watch∗”[All Fields] OR “weight control∗”[All Fields] OR “weight gain∗”[All Fields] OR “weight chang∗”[All Fields]) AND (bloodletting[All Fields] OR blood-letting[All Fields] OR “blood- letting”[All Fields] OR cupping[All Fields] OR ventouse[All Fields] OR venesection[All Fields] OR “spilled blood”[All Fields] OR phlebotomy[All Fields]) AND (“randomized controlled trial”[All Fields] OR “controlled clinical trial”[All Fields] OR “random∗”[All Fields] OR “placebo”[All Fields] OR “trial”[All Fields]

#### 2.3.2 Search for other resources

The reference lists of the selected articles will be read and referred to for a wider literature review. Researchers will also manually search for the offline data.

### 2.4 Data collection and analysis

#### 2.4.1 Study selection

**Figure 1:**
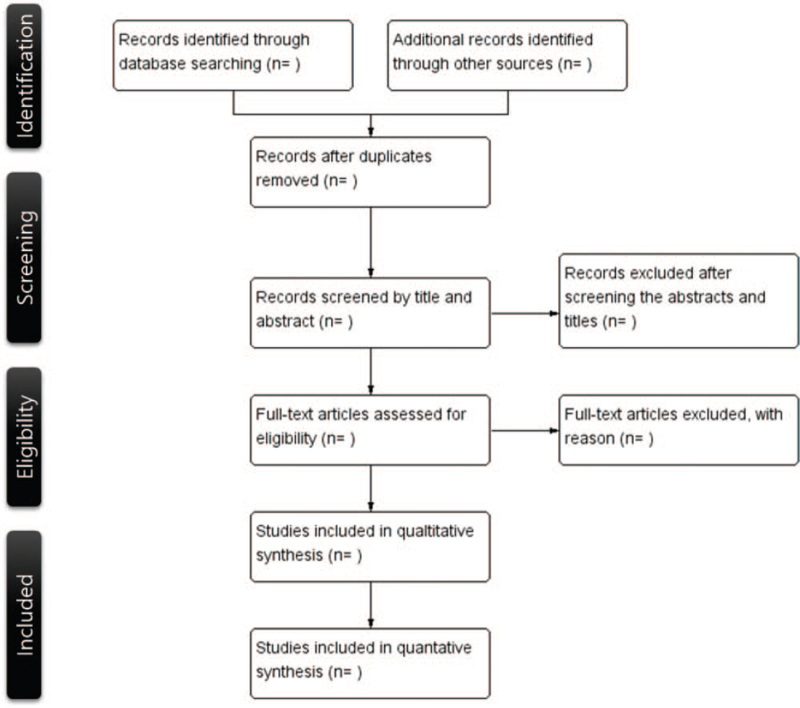
Flow diagram of the systematic review.

Following a singular guideline, 2 reviewers will independently search databases and other sources. The specific contents with examples will be written in the guidelines to ensure better agreement. Disagreements between the 2 researchers will be discussed with a third researcher, and the conclusion of the third reviewer will be the final decision on the issue. A flow diagram of the search and including processes is shown in Figure 1.

#### 2.4.2 Data extraction and management

A common Excel sheet will be shared between the 2 reviewers for data extraction. The items will include the exact information of the authors/ groups; the number of participants; and the details of treatments, such as the type, frequency, and duration, measurements, data of results, and adverse events.

#### 2.4.3 Assessment of the risk of bias and quality of the included studies

The risk of bias will be evaluated by 2 reviewers using the 7 domains stipulated by the Cochrane Collaboration Group.^[11]^ The 7 domains include random sequence generation, allocation concealment, blinding of participants and personnel, blinding of outcome assessment, incomplete outcome data, selective outcome reporting, and other sources of bias. The risk of bias will be graded as either high risk, low risk, or unclear for each domain. If the 2 reviewers disagree on an issue, a third reviewer will make the decision.

#### 2.4.4 Assessment of the effect of treatment

Continuous data will be presented as mean differences and 95% confidence intervals.

#### 2.4.5 Management of missing data

If data are missing in the selected papers, the corresponding author of the article will be contacted for more information. If sufficient data are not provided, the data will not be analyzed.

#### 2.4.6 Data synthesis

When possible, the meta-analysis will be conducted using the Cochrane Collaboration software (Review Manager Software Version 5.3, The Cochrane Collaboration). When *I*^2^ > 50%, a random-effects model will be used, whereas a fixed-effects model will be used when *I*^2^ < 50%. Subgroup analysis will be conducted when the heterogeneity is considered to be high, and the criteria designed by the researchers will be applied when dividing the groups.

#### 2.4.7 Subgroup analysis

The criteria for subgroup analysis will be as follows:

- The type of treatments in the experimental group
- Treatment area
- Duration of interventions

#### 2.4.8 Ethics and dissemination

This is a protocol for a systematic review and meta-analysis. Therefore, no patient information will be included in this study. Ethical approval will not be required for this reason. The results and conclusions of this review will be submitted to a worldwide journal for the dissemination of findings.

## 3 Discussion

Obesity is a prevalent condition in the modern age because of its relationship with other diseases. Cupping therapy is a relatively safe intervention and is known for its effects on various health conditions. Obesity has been treated with traditional Chinese medicine, including cupping, in clinical situations, associated with satisfaction for both patients and doctors. This systematic review will analyze the effect of cupping therapy on obesity. Hopefully, the results of the meta-analysis will be read and applied by clinicians working with obese patients.

## Author contributions

**Conceptualization:** Won-Seok Chung, Koh-Woon Kim.

**Data curation:** Hyungsuk Kim, Koh-Woon Kim.

**Formal analysis:** Hyungsuk Kim, Koh-Woon Kim, Won-Seok Chung.

**Funding acquisition:** Won-Seok Chung.

**Project administration:** Hyungsuk Kim, Koh-Woon Kim, Won-Seok Chung.

**Writing – original draft:** Hyungsuk Kim.

**Writing – review & editing:** Hyungsuk Kim, Won-Seok Chung.
